# SMARTEN: a human-AI hybrid framework for assisted medical literature analysis and its evaluation

**DOI:** 10.1186/s12909-026-08837-0

**Published:** 2026-02-18

**Authors:** Jingyun Wang, Ryan Hodgson, Marie Shigematsu Locatelli

**Affiliations:** 1 Department of Computer Science, Durham University, Durham, UK; 2 Department of Anesthesiology and Intensive Care Medicine, Kochi Medical School, Kochi University, Kochi, Japan

**Keywords:** Knowledge discovery, Topic modelling, Automatic literature review, Cognitive load, Technology acceptance, User satisfaction

## Abstract

**Background:**

For medical professionals, the comprehension and analysis of literature is essential, however, it requires time which could be dedicated to the primary duties of providing care to patients. To address these conflicting needs and the challenge of finding new and appropriate information at the right time during medical literature review analysis, we introduce, to the best of our knowledge, *the first automated framework co-designed with medical professionals*, utilising a *human-AI hybrid approach* for the consolidation of large volumes of literature into manageable, topic-based chunks.

**Methods:**

For this framework (named SMARTEN), on the AI side, cluster-based topic modelling is deployed for the analysis of academic articles (consisting of abstracts and full article content) extracted from the PubMed database, the go-to venue for medical professionals, to ensure the provision of a wide range of topics reflecting this rich field and access to *large volumes* of biomedical literature. After consolidating topics within the literature, semantic analyses of the relationships between topics are performed, based on the content of the literature within each topic, ultimately assisting in the discovery of new knowledge from the literature. The effectiveness of the proposed approach was evaluated through a software implementation of the SMARTEN framework (*the SMARTEN system*), and a subsequent *experimental study* upon the concepts of cognitive load, technology acceptance, and general satisfaction by 18 medical practitioners and medical students. Moreover, the labels assigned by participants to publications were collected and analysed to explore the average accuracy for identifying relevant publications in the SMARTEN system.

**Results:**

Overall, our analysis of user feedback and user logs indicates that the SMARTEN systsem contributed to enhancing the discovery of new knowledge within the literature, while maintaining cognitive load at manageable levels for medical professionals and students. Furthermore, our SMARTEN system effectively identifies relevant publications that would typically appear far down in PubMed search rankings, consistently positioning them within the top 10 results.

**Conclusion:**

This study presents the SMARTEN framework for the automated analysis of large volumes of academic literature based on the semantic contents of individual publications via cluster-based topic modelling. This enables the expediting of literature review for busy medical experts, while also enabling the discovery of new knowledge which would otherwise have gone unnoticed in the wealth of research available online. We evaluate the system developed based on this framework in an experimental study with medical experts, to reinforce the benefit that this framework provides and to identify improvements for further research.

**Supplementary Information:**

The online version contains supplementary material available at 10.1186/s12909-026-08837-0.

## Introduction

Medical professionals need to maintain up-to-date knowledge in a rapidly progressing discipline [1] where new knowledge could be a determining factor in ensuring the identification of a suitable route of investigation when making a diagnosis. However, the exponential growth of human knowledge [2] makes it increasingly difficult to stay current with relevant literature, as individuals often struggle to identify which publications are worth exploring amidst the vast and ever-expanding pool of information—even within a single field. Furthermore, the laborious aspect of manual literature analysis requires that healthcare professionals allocate substantial time to explore a new field with sufficient depth. Consequently, there is a pressing need to streamline the process of rapidly understanding such literature.

To mitigate these challenges, our previous work explored concepts regarding *automatic literature review* through a small-scale preliminary study of literature related to the human kinome [3]. This was achieved by employing a *topic modelling algorithm* to discern themes within literature. Topic modelling can be described as extracting latent semantic structure, commonly referred to as *topics*, within a large collection of documents [4, 5]. Topic modelling presents advantages in text mining tasks, due to the unstructured nature of textual content in academic and medical literature, where *unsupervised learning* approaches are more likely to succeed.

However, for a literature analysis framework to be robust enough for adoption by medical professionals, it must demonstrate the capacity to analyse any literature search defined by the user. Furthermore, such a framework must present information in a manner that effectively supports knowledge discovery while minimising cognitive overload. This balance is essential, as excessive information can reduce the framework’s practical utility if users are unable to process the content efficiently [6–8]. Accordingly, this paper seeks to address the following research questions:

1. *How can cluster-based topic modelling techniques contribute to the design of a visualisation framework that can assist in the analysis of medical literature?*
2. *From the perspective of human-computer interaction, what are the perceptions of medical professionals regarding the use of a software implementation of the proposed framework?*
3. *How does applying the cluster-based topic modelling algorithm Top2Vec* [5] *in the proposed framework, as a method of consolidating literature topics, influence the accuracy of identification of relevant medical papers in practical use cases?*

Our work presents the following contributions:

1. We *co-design essential functions with medical professionals*, to assist with fine-grained literature searches and analysis.
2. We propose the *first comprehensive automatic review framework for medical literature*, able to accommodate the specific needs of medical researchers through a *human-AI hybrid approach*. This *aSsisted MedicAl LiteRaTurE aNalysis* (SMARTEN) framework permits the generation of user-defined queries for analysing different areas and categorising identified literature according to the semantic content of each piece, facilitating filtering by users. Moreover, to mitigate the need for expert intervention, which was required in an existing literature analysis framework [9], the SMARTEN framework adopts cluster-based topic modelling via the Top2Vec algorithm [5]. This ensures automatic detection of topics in the literature, making the overall framework more accessible to users.
3. In addition to the proposed SMARTEN framework, we implemented and evaluated in real-life settings a software implementation (the SMARTEN system). We conducted an experimental evaluation addressing SMARTEN system with 18 medical experts, to explore how the *large volume* of medical literature is consolidated, in a semantically-relevant way; the analysis results of user feedback and user logs confirm the feasibility and practicality of the SMARTEN framework and contribute to highlighting future improvements of the literature assistant.

## Related work

### Current state of literature in automatic literature support

The extraction of insights from vast scientific literature is hindered by its specialised language, which poses significant challenges for conventional keyword-based approaches. However, recent developments in deep learning have enabled the application of contextually aware techniques, which have proven to be effective in managing, organising, and interpreting academic content [10].

More specifically to a semantics-enabled domain, Kilicoglu et al. [10], defined four broad domains, namely *literature-based discovery*, *automated knowledge-base construction*, *knowledge-augmented biomedical natural language processing (NLP)*, and *literature search and information retrieval*. Literature-based discovery techniques are centred on the discovery of literature from structured data within knowledge bases. Examples include the characterisation of the literature discovery as a link prediction task for the production and analysis of a knowledge graph, focusing on Alzheimer’s disease [11]. Automated knowledge-base construction, on the other hand, focuses on the construction of knowledge-bases through the extraction of relations from biomedical literature. This could be through the construction of graphs, encoding relationships between food, medicine, and mental illnesses [12], or the visualisation of *named entities* within the literature [13]. For knowledge-augmented biomedical NLP, the authors categorise works that incorporate knowledge-base derived information, for the improvement of pre-trained language models, or improvement in the performance on specific NLP tasks. From the perspective of pre-trained language models, it has been demonstrated that fine-tuning of language models upon biomedical literature can contribute to state-of-the-art results upon biomedical NLP benchmarks, with language models such as PubMedBert [14], SciBERT [15] and BioBERT [16]. Finally, for literature search and information retrieval, examples include the incorporation of semantic information to improve the retrieval of biomedical literature, by contributing to the queries used during literature retrieval. An example of such works is a method for fine-grained analysis of MeSH concepts, which are commonly used in the semantic indexing of biomedical concepts [17]. Alternatively, Khader and Ensan [18] investigated a Contextual Query Expansion framework, which involved a deep learning-based language model, tasked with the generation of candidate query expansion terms, which may enhance an original query. Based on results from the TREC-COVID dataset [19], the work demonstrated a drastic improvement in search performance when expansion terms were used, compared to the original query. This is of interest, as it indicates that the definition of suitable search queries is vital in ensuring the retrieval of suitable results. We take this into consideration when designing our framework for assisted literature analysis, through the provision of aliases and rule-based logic in filtering search terms prior, and during analysis (see Search and retrieval section).

### Topic modelling in assisting the literature review process

The application of machine learning (ML) or artificial intelligence (AI) for knowledge synthesis is increasingly advanced, yet the selection of an appropriate method remains contingent on the specific research goal. In classification tasks, where a prior goal is known and labelled data exists, supervised learning is used to train a model to classify new data. Works using this approach targeted, for example, the classification of medical literature based on hallmarks of cancer [20] and cancer susceptibility genes [21]. This falls into the domain of supervised learning. In contrast, for knowledge discovery in academic literature, an investigator may have no prior knowledge of a topic and therefore seek to identify patterns or trends within data, without previously labelled data. In such tasks, unsupervised learning techniques are better suited. Soldaini and Goharian [22] presented an unsupervised method for the extraction of named medical entities from medical literature. Similarly, Ding and Luo [23] presented a neural network attention-based approach for unsupervised extraction of keyphrases from medical literature, which can then be provided as a visualisation of relevant keyphrases in a knowledge graph format, based on a given user query.

As an unsupervised learning technique, topic models were originally introduced as a probabilistic modelling technique for the identification of semantic structure of a corpus, based on a Bayesian analysis of texts [24–28]. Latent Dirichlet Allocation (LDA) [24], is a well-known variant of a topic modelling algorithm and introduced a generative probabilistic model, based on the hypothesis that documents can be represented as random mixtures over latent topics, with each topic entailing a distribution of words. Since its inception, LDA has been successfully applied in several research domains, such as topic modelling of scientific abstracts [24, 25], detection of hate speech [29, 30], and opinion mining [31], to name a few. Specifically, LDA and derivatives based on it, have been applied in several works contributing to medical and biomedical domains. Bio-LDA [32] demonstrated that LDA can be applied to the identification of biological terminology within PubMed articles and to identify latent topics within the literature. From a more general perspective, [9] adopted LDA for knowledge discovery in the academic literature, through the proposed “Smart Literature Review” framework to identify topics within the literature.

There are, however, limitations to using LDA for automated literature analysis [24]. LDA requires a prior specification of the number of topics to be extracted from the literature, and the correct specification of this hyperparameter is essential in identifying meaningful topics from the corpus. Methods to mitigate this exist, such as evaluating topic coherence metrics across a range of topic numbers, as used in [9]; however, this is an exhaustive and time-consuming approach that makes it unsuitable for when results must be provided in a practical amount of time. Additionally, pre-processing steps are required, to remove frequently used words within the literature, which do not contribute any semantic meaning, commonly referred to as stopwords. In brief, LDA is a bag-of-words approach to topic modelling, and any literature analysis based on this algorithm cannot account for the contextual structure of documents.

In comparison, the Top2Vec algorithm [5] provides an alternative to Bayesian topic models such as LDA [24], eliminating the need for pre-defining the topic number and filtering stopwords. This is achieved by leveraging pre-trained language models, such as Doc2Vec [33], Bidirectional Encoder Representations from Transformers (BERT) [34], or Universal Sentence Encoder [35], to produce a vector representation of each document within the corpus. A semantic embedding of joint document-word vectors is computed where the distance between document and word vectors represents semantic association. This ensures that semantically similar documents achieve a smaller distance between each other, compared to dissimilar documents. Document embeddings are then clustered using the Hierarchical Density-Based Spatial Clustering of Applications with Noise (HDBSCAN) [36] algorithm, with resulting cluster labels representing the topic of each document within the corpus. The hierarchical nature of HDBSCAN ensures automatic identification of the topic number, which eliminates the exhaustive evaluation of topic numbers necessary in the framework presented by [9]. In this work, we refer to this approach as *cluster-based topic modelling*, based on the assumption that topics can be represented as dense regions of text embeddings identified via clustering algorithms. Top2Vec has been demonstrated to outperform LDA when applied to the 20NewsGroups [37] benchmark dataset [5]. Alternative algorithms, such as BERTopic [38], operate under the same overarching assumption. However, BERTopic introduces class-based TF-IDF (c-TF-IDF) for the identification of topic words by aggregating all documents within a cluster into a single pseudo-document and subsequently extracting the most frequent terms from this aggregated representation. This procedure has been shown to enhance the interpretability of the resulting topic terms. In summary, based on the aforementioned advantages over LDA, we have selected Top2Vec as a suitable topic modelling algorithm for the identification of research topics within the literature, which ensures to mitigate the need for researcher intervention during the topic modelling process.

## Methodological design of the SMARTEN framework

**Fig. 1:**
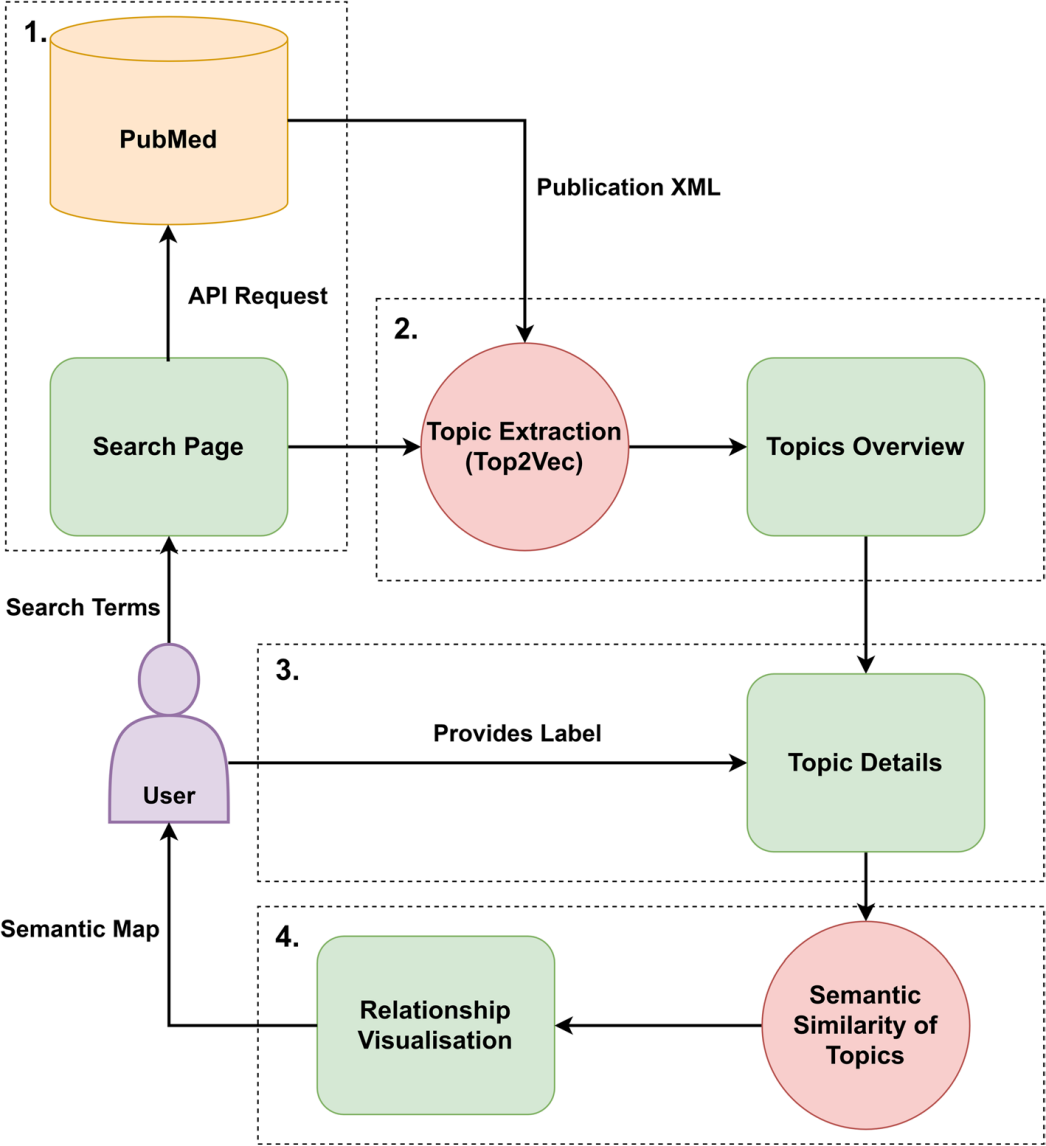
The stages of the SMARTEN framework

To address the first research question, building on our previous work on the visualisation of topics in the literature identified by cluster-based topic modelling [3] , we engaged with medical experts to identify several aspects of usability and visualisation that were specified as beneficial to any downstream analyses required by medical experts, these are presented in Co-design of the SMARTEN framework section. The overall structure of the SMARTEN framework is presented in SMARTEN framework for biomedical literature section, and visually represented in Fig. 1. In addressing the second research question, we focus on an experimental evaluation of the overall framework, through a software implementation of the proposed framework, and a subsequent in-depth evaluation study involving specialist users from the medical domain. The experimental design is detailed in Experimental design section. Finally, to examine the third research question, we assess the accuracy of the SMARTEN framework, by evaluating the labels assigned by study participants, compared to the number of papers recommended by the framework. The method for this is detailed in Assessment of relevance of identified publications section.

### Co-design of the SMARTEN framework

A co-design methodology was adopted in designing the SMARTEN framework, to ensure that direct benefit could be made to medical experts. This co-design process consisted of several stages of discussion, with feedback provided by medical experts contributing to design changes. This was followed by a wider study of the effects of design choices. For the first part of this process, we conducted the co-design discussion with a medical expert, Key Opinion Leader (KOL) of the experimental focus group, and co-author of this paper. The medical expert liaised with colleague doctors and medical students based on their evaluation of the system development and identified functionality which they requested or modified. In general, this co-design approach closely resembled a SCRUM software development method [39].

#### Accounting for synonyms during literature search

**Fig. 2:**
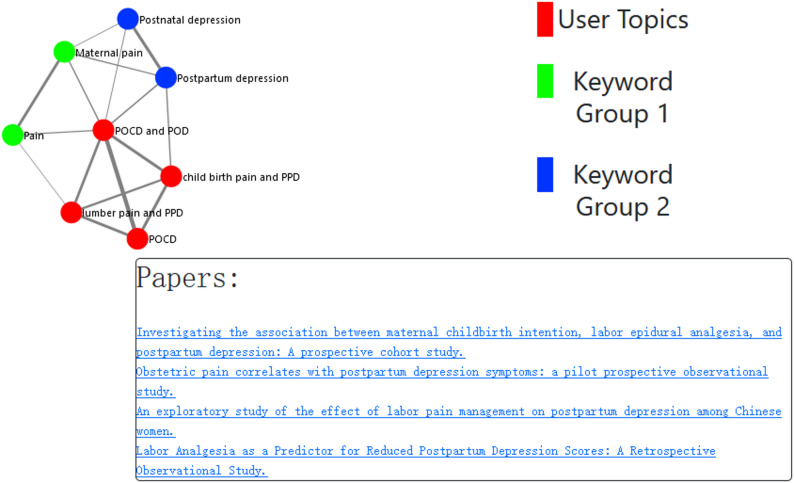
The graph view within SMARTEN, showing topic relationships based on semantic similarity

Amongst outcomes, the medical expert underscored the advantages of implementing a *multi-keyword search capability*. In the medical domain, one keyword may have several synonyms; therefore, it is important to provide a function that allows the user to specify the aliases of each keyword. This was taken into consideration when designing the framework, ensuring that users may provide search terms in groups, so that within each group, multiple synonyms (or aliases) can be provided. For example, a user may wish to create a search group consisting of a keyword *“Maternal Pain”*, and its alias *“Pain during pregnancy”*. An additional outcome during co-design was the expressed desire of medical professionals to amalgamate these alias groups, facilitated through logical operators. Based on the criteria provided by the user, a query can be constructed using logical operators when querying PubMed literature. In the case above, if the user creates another keyword *“Postpartum depression”* and its alias *“Postnatal depression”*, the final request could be extended to: *“(Maternal Pain OR Pain during pregnancy) AND (Postpartum depression OR Postnatal depression)”*. Users can enter these query terms when using tools developed based on the SMARTEN framework. Although this functionality is already provided by some online literature search platforms, such as the PubMed web portal, we extend this functionality through subsequent analyses, by automatically consolidating large amounts of literature into topics and allowing analysis of semantic relationships of the aliases provided by users and comparison of these with topics identified within the literature, as shown in Fig. 2. We provide a deeper explanation of this functionality in Semantic similarity of identified topics section.

A key negotiation emerged regarding the scope of this feature. The KOL initially envisioned a system capable of handling searches across three or more distinct keyword groups simultaneously, considering this a ’must-have’ for complex clinical querying. However, the development team raised concerns that the computational complexity of comparing N number of groups (an N-wise comparison) would scale exponentially, requiring immense infrastructure and threatening the project’s feasibility within its intended timeline and budget.

This conflict was resolved through a series of iterative dialogues and prototyping. The development team built a prototype demonstrating the performance and interface implications of supporting two groups versus three or more. This tangible demonstration allowed for a productive negotiation: the KOL and their peers recognised the disproportionate resource investment for a feature that would be used less frequently. A consensus was reached to limit the initial implementation to two keyword groups, prioritising a robust and performant core feature set. This feature was strategically designed to be extensible, with the option to scale to more groups in a future version pending additional resources and user demand.

This example provides a critical practical lesson for co-design: Prototyping is an invaluable tool not just for design, but for feasibility negotiation. A functional prototype can translate abstract technical constraints into concrete user experiences, enabling clinical experts to make informed trade-offs between ideal functionality and practical implementation, ultimately leading to a more viable and focused product.

#### Identification of meta-information within publications

During co-design, we introduced various types of information that could be gleaned from publication metadata, such as publication keywords, Medical Subject Headings (MeSH terms), year of publication, authors and named chemicals or medicines discussed in a publication. Subsequently, the medical expert identified which of these data points would enhance their analytical processes. For this, the expert identified the presence of *MeSH terms, chemicals, publication keywords, and year of publication* as being beneficial in contributing to their understanding of topics identified within the literature.

#### Defining the number of publications to return

It was highlighted during co-design that providing a high number of publications can overwhelm users, making it difficult to identify publications that would be useful in their search. Thus, one of the outcomes of co-design was the definition of providing only a *limited subset*, to retrieve the ten most relevant publications identified as belonging to each topic.

### SMARTEN framework for biomedical literature

Here, we define an overall structure for the framework, broken down into four distinct stages, which are numerically labelled in Fig. 1. In the first stage, users provide search terms via the search interface, which allows the provision of keyword groups, as detailed in Search and retrieval section. These search terms are structured into an API request for the PubMed API, to retrieve all publication data in a structured eXtensible Markup Language (XML) format. Secondly, the response for this API query is parsed, with the full-text and metadata for each publication being extracted. Publication texts are then analysed using a cluster-based topic modelling algorithm (the Top2Vec [5] algorithm is used in our study), with an overview of the topics identified within the literature then being presented to the user via the topic overview screen. At the third stage, a user may view publications which have been assigned to each topic on the topic details page, along with topic metadata consisting of the frequencies of keywords, MeSH terms, chemicals, and volume of publications based on publication date, which were defined during the co-design process. Based on this information, a user may label publications as relevant to their search focus. Finally, the fourth stage entails the processing of the labelling provided by a user into a graph network-based visualisation (as shown in Fig. 2), based upon the semantic similarity of the publications labelled by the user.

## Design and evaluation of SMARTEN system

**Fig. 3:**
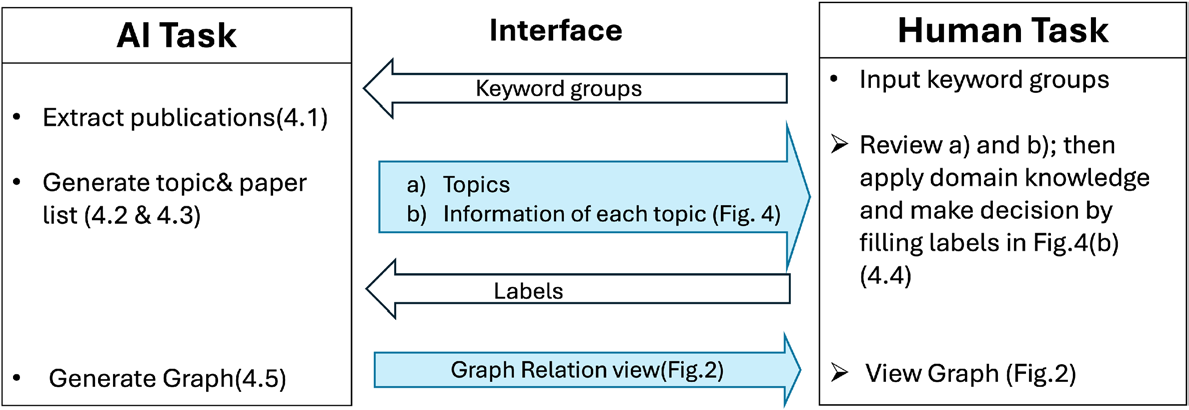
The hybrid human-AI workflow in SMARTEN

**Fig. 4:**
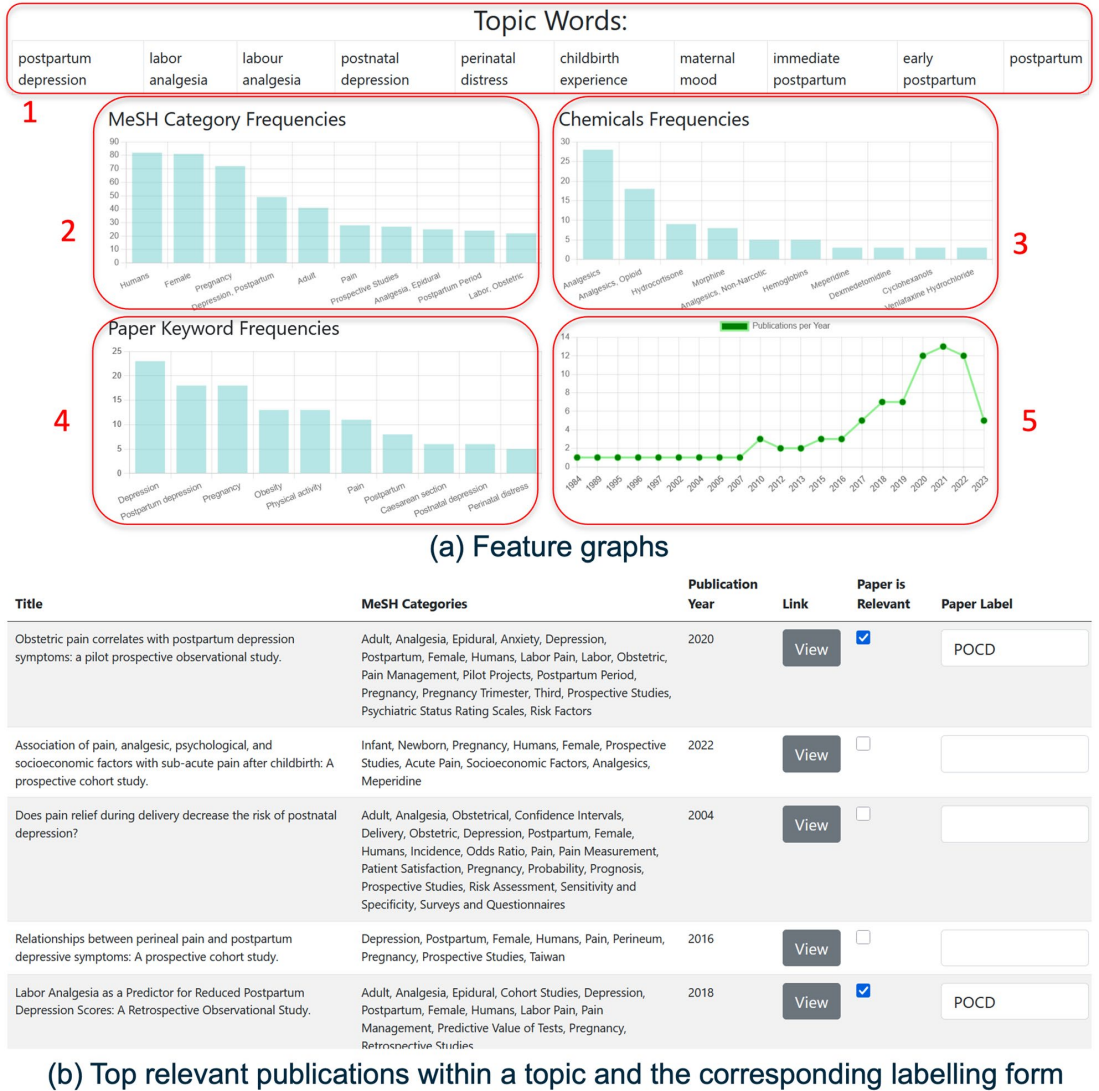
The topic information interface of the SMARTEN system, visualising (**a**) Feature graphs, and (**b**) Top relevant publications within a topic and the corresponding labelling form.

Based on the defined SMARTEN Framework, we develop the SMARTEN system, transforming all the requirements of the framework into its concrete features. Figure 3 demonstrates the human-AI hybrid workflow in the SMARTEN system. In each iteration, the medical expert is presented with a list of candidate topics proposed by the AI model. For each candidate topic, the SMARTEN interface presents the expert with the top 10 papers most relevant to the search terms, alongside several key visual features, as shown in Fig. 4. In the experiment of this study, the expert’s task was to evaluate the relevance of each paper by synthesising the AI’s output with their domain knowledge. Based on this synthesis, the expert applied a binary label (by clicking the ‘Relevant’ box) and provided a written description for each paper deemed “Relevant”. Eventually, this labelled data served as explicit feedback for the AI system. The AI sytem used this feedback to refine its topic model and subsequently generate an updated knowledge graph (Fig. 2), which now more accurately reflects the expert’s judgment. This graph was then presented to the user, completing one iteration of the hybrid loop.

### Search and retrieval

As defined in Accounting for synonyms during literature search section, search terms created by users are provided as a query to the PubMed repository through Entrez E-Utility [40]. Publication results are returned in a structured XML, which includes textual content and metadata associated with publications (a full summary of the available data can be found at [41]). Extraction of publication XML is used to identify the title, text content, publication data, MeSH terms, keywords, and chemicals associated with each publication which is used in the subsequent analyses.

### Topic modelling of identified literature

Next, reflecting stage 2 of the framework proposed in SMARTEN framework for biomedical literature section, the identification of topics from within the literature is performed using the Top2Vec algorithm [5]. As discussed in Topic modelling in assisting the literature review process section, publication title and text are first converted into an embedding encapsulating the semantics of the text in a highly-dimensional vector via a language model. These highly dimensional representations cannot easily be used in downstream tasks such as clustering, as downstream algorithms may be negatively affected by the “curse of dimensionality”. Therefore, dimensionality reduction is performed through the Uniform Manifold Approximation and Projection (UMAP) [42] algorithm, which reduces highly dimensional document embeddings into low-dimensionality representations, while preserving global and local features of the embeddings. For clustering, Top2Vec applies the HDBSCAN algorithm [36], which performs hierarchical clustering based on the density of the vectors produced by UMAP [42], with resulting clusters representing topics identified in the literature. Thus, the ranking of papers listed in each topic is based on the cosine-similarity of the document vector to the centroid of their assigned cluster.

Users then view detailed information on a particular topic identified within the literature, as shown in Fig. 4a. This provides users with metrics to assist in their comprehension of literature within the topic, defined based on feedback during co-design (Identification of meta-information within publications section): This includes: 1. A breakdown of the general keywords that can be used to describe the topic. 2. A bar chart, representing the frequency of Medical Subject Headings (MeSH) categories. 3. The frequencies of individual chemicals or medicines. 4. The frequencies of publication keywords. 5. The frequency of publications within the topic based on year of publication.

### Limiting the publication search

Additionally, regarding the meta-information identified in Defining the number of publications to return section, the SMARTEN system provides users with a view of the 10 most relevant publications that reside within a topic (Fig. 4b), where the degree of relevance is determined based on the cosine similarity distance of the individual publication embedding, relative to the cluster centroid, which determines the central embedding of the topic.

### Meta-information and manual labelling

Furthermore, regarding the meta-information identified in Identification of meta-information within publications section, as well as stage 3 of the framework (SMARTEN framework for biomedical literature section), as shown in Fig. 4, users have the option to view the full-text version of the paper, as well as see the title and MeSH categories for individual publications. Based on the information provided, users can then choose to label individual publications as being relevant to their literature search and assign a label of their choosing to any relevant literature.

### Semantic similarity of identified topics

Finally, as defined in the requirements in Accounting for synonyms during literature search section, and reflected in framework stage 4 in SMARTEN framework for biomedical literature section, relevant literature identified by users can be analysed and visualised in terms of the semantic similarity of publications that have been labelled by users. Figure 2 presents an example of the general view of this visualisation in the SMARTEN system, while the expanded list of publications assigned to a topic “POCD” is displayed after its representing node is clicked. In this relationship graph of topics, nodes are colour-coded to represent the topics identified by users in red, search terms from the first alias group in green and the second alias group in blue.

For this relationship graph, the SMARTEN system groups individual papers by the labels assigned by the user and then calculates an overall *topic vector* taken as an average of the individual embeddings for each paper in the topic group. All combinations of topic vectors are compared based on cosine similarity to calculate their distances. The cosine similarity distance measure represents the distances between two topic vectors, with a higher similarity demonstrating that two groups of documents are closely related. Once similarities have been calculated for all combinations of topic vectors, a *network graph* is created, by considering the top three highest-ranking connections for each topic. The search terms provided by the user when initiating the literature search are included as nodes on the graph, which are generated through the same embedding method by which the papers were embedded, such that they can be compared using cosine similarity. Once constructed, the graph demonstrates the degrees of similarity between topics and the user’s search terms. Additionally, this graph is used in SMARTEN system to allow users to view the papers they have assigned to each topic, by directly clicking on individual graph nodes.

This graph view was designed to demonstrate to the user how the topics that they have deemed relevant to their investigation relate to each other, and the search criteria that they adopted. This is intended to assist in the comprehension of topics that may be new knowledge to the user, and the effects of this are analysed as part of our study.

### Experimental design

To evaluate the effectiveness of SMARTEN, it is important to ensure a suitable cross-section of the target user group, which are people with medical backgrounds. Participants were invited from the Department of Anaesthesiology at a university-affiliated hospital in Japan. The inclusion criterion mandated that all participants must be actively engaged in clinical anaesthesia or perioperative care, ensuring relevance to the intended use case of the tool. This department was selected for both feasibility, given a co-author’s affiliation, and to provide a controlled, homogeneous clinical environment for a specialised analysis of system accuracy, minimising cross-disciplinary variability.

Recruitment was successful, resulting in a final cohort of 18 participants. This cohort provided a suitable cross-section of the medical hierarchy, comprising: 2 Medical Students on clinical placement, 5 Junior Resident Doctors, 6 Senior Resident Doctors, 4 Staff/Consultant Anaesthetists, and 3 Medical Researchers with clinical duties. The group had a relatively even gender distribution (10 females, 8 males) and an age range spanning from 18 to 50 years. The environment of intended use mirrored their professional roles: the tool was evaluated in the context of clinical practice for practitioners and during clinical placements for students. This demographic diversity ensures that the evaluation findings are grounded in the perspectives of a broad spectrum of the system’s potential end-users. While the sample size (N=18) may be considered a limitation for broader generalisation, it is consistent with many user studies in clinical settings where recruiting busy healthcare professionals is a significant challenge.

#### Experiment briefing

Participants were briefed on an overview of the purpose of the experiment and SMARTEN tasks. The ethical procedure is detailed in section “Ethics approval and consent to participate”. Following this briefing, system access was provided to participants, enabling the collection and storage of user interaction logs, including literature searches, topics identified, and the labels assigned by users to topics and publications. Participants were not given any subject on which to base their literature search and were encouraged to use SMARTEN system to investigate a domain of their choosing. Once the search terms are entered, the SMARTEN system would present participants with a list of the topics found in their literature, generated through the topic modelling process. Users may then choose to analyse individual topics and provide labels to any publications identified within the wider literature search which they felt were relevant. Subsequently, participants could view the relationships of the topics they identified, along with their original search terminology (as presented in Fig. 2), based on the semantic similarities between the topics and search terms.

#### Participant questionnaire

After completion of the literature analysis task, participants were presented with a seven-point Likert-type scale questionnaire designed to gauge their perceptions of the interaction with the SMARTEN system. The questionnaire was structured around three key constructs: *cognitive load* [6], *technology acceptance* [43], and *general satisfaction*. These constructs were selected based on their relevance to evaluating user interaction and system usability in technology-enhanced learning contexts. The items were adapted from previously validated scales in our earlier work [44–47] and further refined.

*Cognitive load* was conceptualised in terms of *mental effort* and *mental load*, consistent with established cognitive theory frameworks [6–8]. *Mental effort* reflects the cognitive capacity required to understand the purpose of the experiment and to complete tasks using the SMARTEN system; *mental load* refers to the cognitive demands imposed by the task, including perceived distractions and stress encountered during system use. This construct was operationalised using four items: two measuring mental effort (understanding task goals and completing the literature analysis) and the other two measuring mental load (perceived distraction and stress during the task). Responses were collected using a 7-point scale (1 = Not at all, 7 = Very high cognitive load). Full item wording is available in Appendix Table A1.

*Technology acceptance* was defined as participants’ perceptions of the system’s usefulness and ease of use, aligning with the Technology Acceptance Model [43]. Two items were used: *perceived usefulness* and *ease of use*. Responses to three main system functions (feature graphs, publication labelling, and relation maps) were recorded on a 7-point scale ranging from 1 (Strongly disagree) to 7 (Strongly agree), with 4 indicating neutrality. The exact item wording is provided in Appendix Table A2.

*General satisfaction* was defined as the users’ overall subjective evaluation of how well the SMARTEN system supported their academic and professional needs, especially in the context of medical literature review tasks. This construct was measured using a customised 10-item scale developed based on prior validated satisfaction instruments [44–47], adapted to the study’s specific context. Full items are available in Table 3. At the end of the questionnaire, participants were invited to provide open-ended comments regarding their experience using SMARTEN and suggestions for improvement. These qualitative responses were used to contextualise and enrich the quantitative findings.

#### Assessment of relevance of identified publications

Further to responses recorded by the questionnaire, we analysed the labels assigned by participants to publications to explore the average accuracy of applying Top2Vec to identify relevant publications in the SMARTEN framework, addressing the third research question. We calculate the percentage of the papers which the user has assigned a “*relevant*” label, relative to the total number of papers suggested to the user. As the Top2Vec model may suggest a large number of topics to the user, we only count topics which have been assigned at least one “*relevant*” label by users, as we deduce that any topics that have not been accessed or labelled by the user are not relevant to their investigation. We present the findings of this analysis in Analysis of user labelling section.

## Results

### User perception analysis

As defined by research question RQ2, we are interested in the user perception of their cognitive load, technology acceptance, and general satisfaction. Table 1 displays the outcomes of evaluating the cognitive load of the 18 participants while engaging in the literature analysis task. In terms of *mental effort*, the median rating for the perceived *“effort required to understand the activity’s purpose”* was 3.5 ($IQR = 3$), suggesting a typical experience of moderate effort. However, the high IQR indicates significant variability in user experience. The median score for the *“effort required for labelling the papers”* was 5.00 ($IQR = 1.75$). This indicates that the task was generally perceived as more challenging, though the considerable IQR reflects notable variability in participants’ opinions. In terms of *mental load*, the median ratings of the degree of *“distraction”* and *“stress”* while using the SMARTEN system were both around 4 ($IQR=2.75$); suggesting that users typically experienced moderate levels of distraction and stress, though opinions on this varied considerably.

**Table 1:** Cognitive load of the literature analysis activity in the SMARTEN system (1-7, 1: not at all, 7: very high)

	Mental Effort		Mental Load	
	Purpose	Labelling	Distraction	Stress
Median	3.50	5.00	4.00	3.50
IQR	3.00	1.75	2.75	2.75

In terms of *technology acceptance*, as shown in Table 2, analysis of *“perceived ease of use”* indicates high usability across all three functions: feature graphs analysing (Fig. 4a), publication labelling (Fig. 4b), and relation maps (Fig. 2). The median rating for each was 6 ($IQR = < 1$), demonstrating strong participant agreement that the tools were straightforward and easy to get accustomed to. Median ratings of *“perceived usefulness”* of these three functions are all equal or greater than 6 ($IQR=1$), which implies that most participants agreed that these three functions were useful for improving their literature analysis.

**Table 2:** Technology acceptance of the SMARTEN system (1-3: strongly to slightly disagree, 4: neutral, 5-7: slightly to strongly agree)

	Feature Graphs		Publication List Labelling		Relations Map	
	Easy	Useful	Easy	Useful	Easy	Useful
Median	6.00	6.00	6.00	6.00	6.00	6.50
IQR	0.00	1.00	0.00	1.00	1.00	1.00

When asked about the feature graphs (*MeSH Category Frequencies*, *Chemicals Frequencies*, *Paper Keyword Frequencies*, and *Publication per year*) that may not have been beneficial for their analysis and could be excluded (where participant experts could select from multiple choice options), half of the participants opted for none, 7 participants selected MeSH Category Frequencies, and 4 participants opted for Chemicals Frequencies. This suggests that the MeSH Category Frequencies and Chemicals Frequencies may be less valuable, compared to the other categories.

Table 3 presents the median ratings for the 10-item general satisfaction questions. All items received a median rating of 6. This indicates that the vast majority of respondents were highly satisfied with the system’s functionality. Most items showed strong consensus ($IQR <= 1$), with the exception of Q5 ($IQR = 1.5$), suggesting a slight lack of consensus on whether the system provided the optimal quantity of information.

**Table 3:** General user satisfaction in the SMARTEN system (1-3: strongly to slightly disagree, 4: neutral, 5-7: slightly to strongly agree)

	Question	Median	IQR
Q1	The system helped me to have a deeper understanding of these topics.	6.00	0.75
Q2	Using the system can help me find new information within the literature.	6.00	1.00
Q3	Overall, the system was easy to use and navigate.	6.00	0.00
Q4	I like to use the system.	6.00	0.00
Q5	Overall, the right amount of information was given by the system.	6.00	1.50
Q6	I felt confident using the system to improve my understanding of new medical subjects.	6.00	0.00
Q7	Using this system would enhance the effectiveness of my literature analysis.	6.00	0.00
Q8	I would be able to use the system without assistance from others.	6.00	0.75
Q9	I would like to use the system in my future work.	6.00	1.00
Q10	I would recommend this system to my colleagues.	6.00	0.00

Notably, Q3, Q4, Q6, Q7, and Q10 achieved perfect consensus ($IQR = 0$). These results indicate users unanimously found the software easy to navigate and use, enjoyed using it, felt confident in its ability to improve their understanding of new medical subjects, affirmed that it enhances their literature analysis effectiveness, and would recommend it to others. Furthermore, the strong consensus on Q1 and Q8 (IQR = 0.75) indicates that the participants agreed that the system facilitated deeper topic understanding and could be used without external support. Overall, the results demonstrate a high level of user satisfaction and a strong perception that the system effectively enhances literature analysis and supports learning in medical domains.

### Analysis of user labelling

The participants were encouraged to label 1 or 2 groups of the topics that they considered to be most relevant to the associated key phrases during their literature search. Table 4 presents the percentage of papers that were labelled relevant by the participants, out of the total number of papers (10 per topic) suggested to them, after topic modelling with Top2Vec. This can be inferred as ‘User-Agreed Relevance’. The lowest User-Agreed Relevance of 15%, suggests that at a minimum, at least 1 out of the 10 papers within a topic was relevant to the participant’s search. Moreover, for 9 out of 18 groups of search terms (IDs 3,5,10, 11, 13-17), the recommendation result based on Top2Vec attained over 50% User-Agreed Relevance, suggesting that these participants believed more than half of the suggested papers were relevant to their research interests. Generally, we argue that an average of $52.3\%$ of relevant articles suggested on relevant topics is beneficial for the discovery of new knowledge and contributes to assisting medical experts in finding new information. It is worth noting that the inclusion of a secondary keyword group is not mandatory, resulting in some users (such as user ID 2) opting to exclude this when their research interest is confined to a specific research focus.

**Table 4:** Search terms applied by the users and the percentage of papers they labelled as relevant

ID	Keyword Group 1	Keyword Group 2	%
1.	Maternal pain, Pain during pregnancy	Postpartum depression, Postnatal depression	16.6%
2.	POCD, Postoperative cognitive disorder, Postoperative cognitive decline	/	40%
3.	Implanted cardio defibrillators	Deactivation	65%
4.	Hypervolemia	Cardiac Surgery	15%
5.	Urinary catheter discomfort	Postoperative	65%
6.	Sevoflurane	Ondansetron	35%
7.	Acute lymphoblastic leukemia	Pediatric	33.3%
8.	Microbiome	Brain-Gut	43.3%
9.	Ammonia	Urinary tract infection	25%
10.	Transcranial magnetic stimulation	Pain	56.6%
11.	Dissection	Bovine	80%
12.	Propofol	Remimazolam	40%
13.	Liver Failure	Acidosis	56.6%
14.	PROM, Preterm premature rupture of the membranes	Antibiotics	50%
15.	Ondansetron	Metoclopramide	100%
16.	Vital Sign	Bleeding	90%
17.	Pancreatoduadenectomy	Fluid	100%
18.	Heart, Coronary	Coronavirus, COVID	30%
**Average**			52.3%

**Fig. 5:**
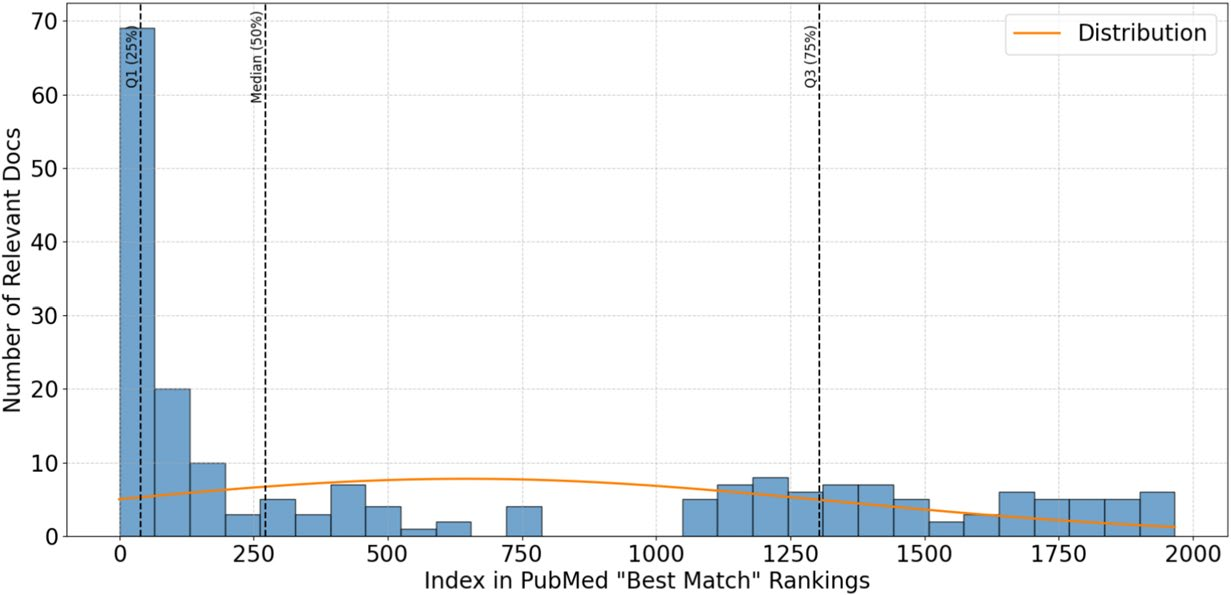
Distribution of relevant labelled publications by users of SMARTEN, by their ranking in a PubMed online search using “Best Match” ranking

Following the extraction of labels assigned by participants to the publications, we explore their location in the conventional rankings of a PubMed online search employing the “Best Match” ranking. The findings of this analysis are outlined in Table 5 and Fig. 5. As illustrated in Table 5, the mean and median rankings of the identified papers vary considerably between both SMARTEN and PubMed, indicating that our system could be advantageous in spotlighting underrepresented research, which would typically be ranked much lower in a traditional search and thus overlooked. Nonetheless, the histogram representation in Fig. 5 indicates that, more than half of the relevant papers ranked after the 250th in PubMed “Best Match.” This suggests that the SMARTEN system is effective in enabling users to discover publications which they would normally have missed in a literature search in PubMed.

**Table 5:** The number of papers analysed by SMARTEN, compared to those identified by a PubMed search, detailing the Mean and Median positions of the User-Assigned Relevant Labelled Publications, based on a PubMed “Best Match” search

ID	SMARTEN	PubMed		
		Total papers	Mean position of relevant papers	Median position of relevant papers
1.	402	605	292.54	243
2.	1,861	6,077	743	743
3.	113	166	47.88	41
4.	68	76	24.33	22.5
5.	156	222	50.1	43.5
6.	49	60	93.53	88.5
7.	1,269	17,070	495	498
8.	714	815	409.15	306
9.	67	127	53.57	63.0
10.	1,188	1,789	490.83	524.5
11.	554	1,847	353.75	118.5
12.	98	371	83.82	80
13.	857	1,042	440.29	420
14.	471	511	162.44	178.0
15.	554	630	687.67	713.0
16.	1,351	12,113	120	120
17.	418	536	86.0	51
18.	1,483	1,882,630	50.86	67.0

In addition to the analysis of face-value relevance and in comparison to PubMed rankings, we examine the influence of keyword specificity on the relevance of search results. Specifically, we analyse the relationship between the proportion of relevant papers retrieved and both keyword specificity and the number of retrieved results. Keyword specificity is estimated by the average length of keywords used in each search, measured in number of characters, while the number of retrieved results is derived from the corresponding PubMed queries.

**Fig. 6:**
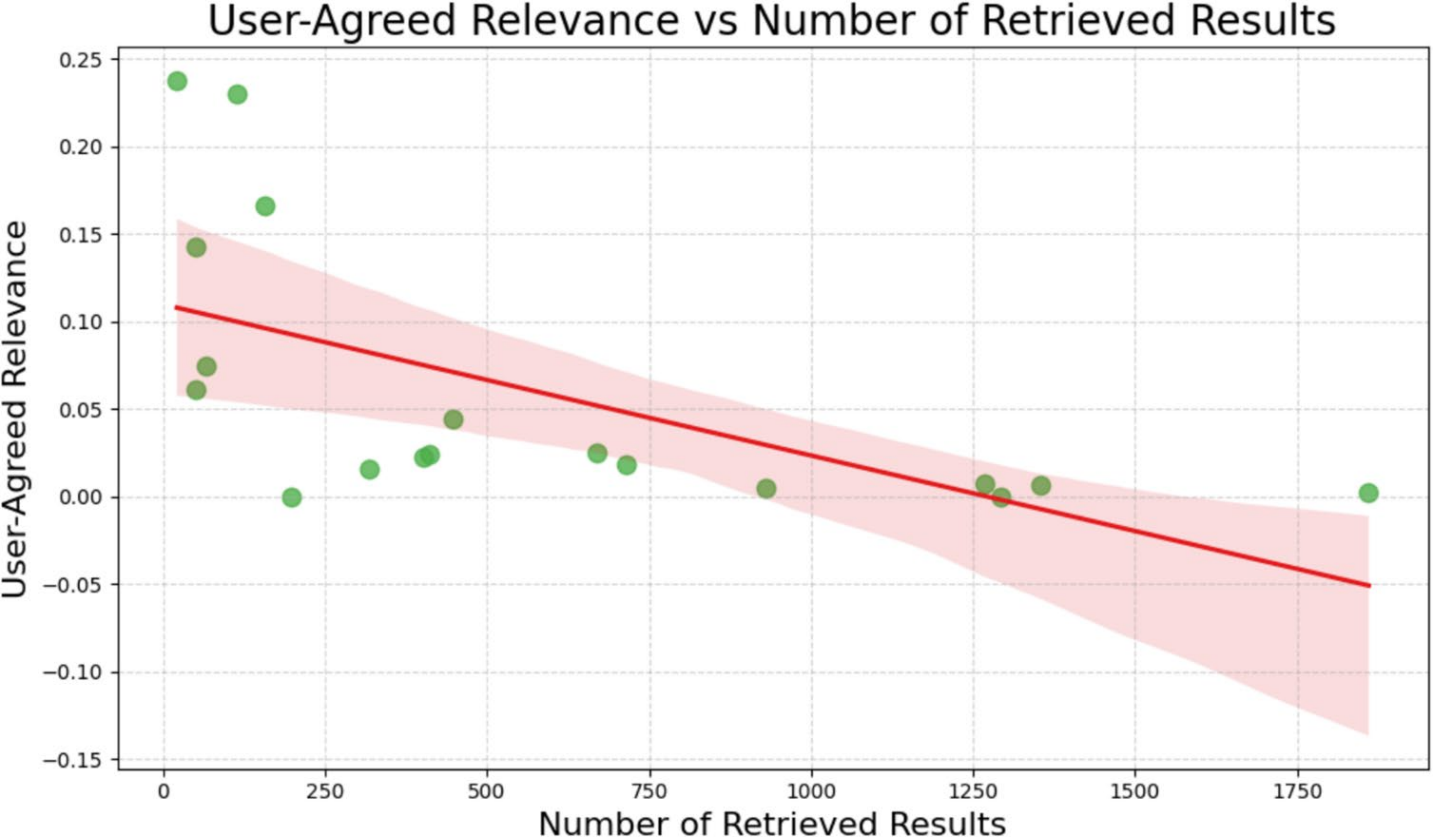
Influence of number of retrieved results on proportion of relevant papers

**Fig. 7:**
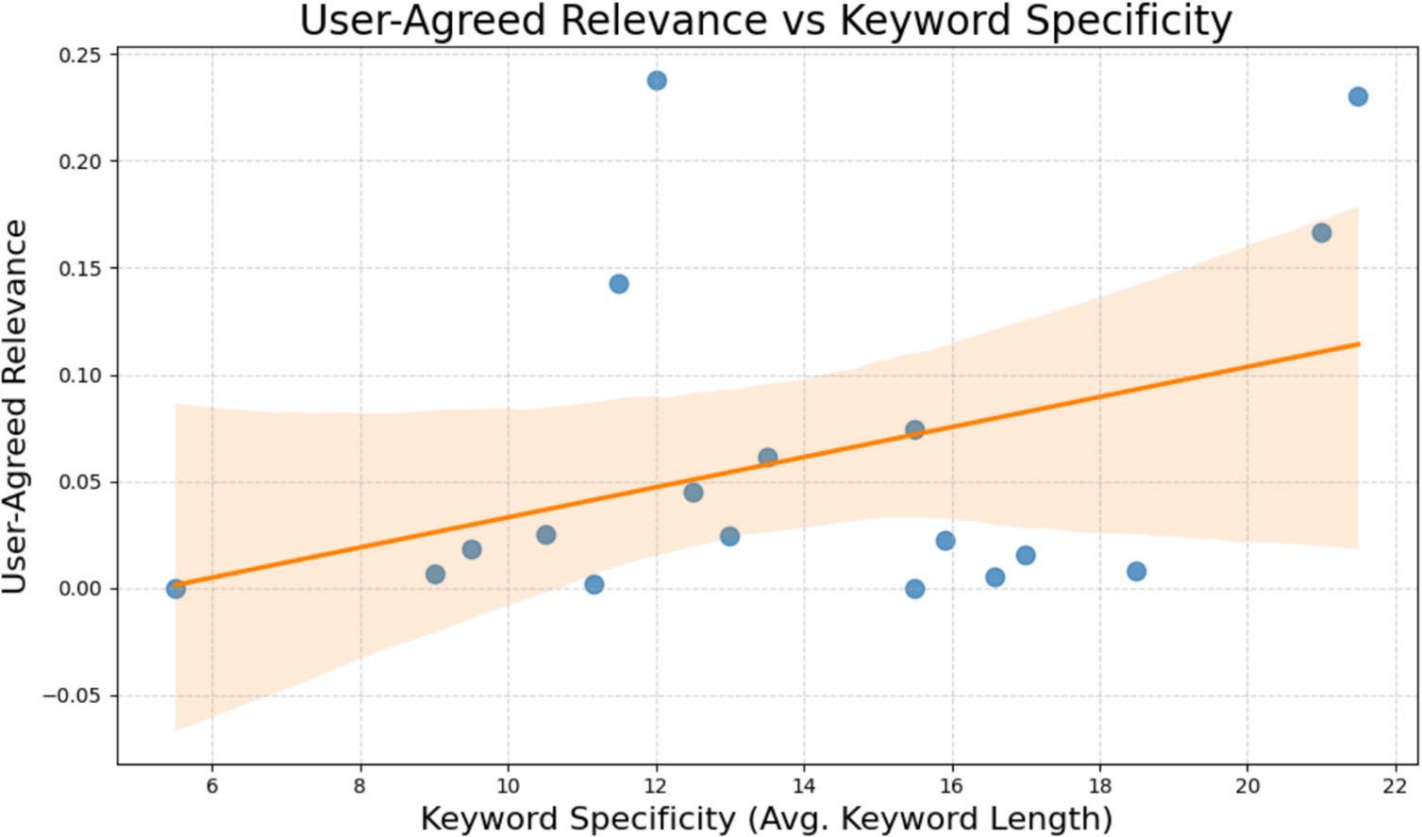
Influence of keyword specificity results on proportion of relevant papers

The findings (Fig. 6) indicate that a smaller number of retrieved results tends to correlate with an improved User-Agreed Relevance, suggesting that queries which yield larger sets of documents tend to dilute the proportion of relevant items among the top-ranked results. Nonetheless, the correlation between the character length of keywords and their specificity is weak, as shown in Fig. 7. This suggests that the number of retrieved results exerts a greater impact on User-Agreed relevance than keyword specificity based on average length.

Crucially, these results suggest that the initially observed correlation between keyword specificity and relevance labelling may be partially attributable to the number of retrieved results, rather than to a direct causal effect. Narrower, more specific queries naturally retrieve fewer results (such as those with an average length greater than 20 in Fig. 7), thereby increasing the likelihood that relevant papers appear in the top positions and receive relevance labels. In contrast, broader queries produce larger result sets, posing challenges to both the ranking algorithm and the participants’ labelling capacity.

It should be noted that the topic modeling algorithm applied in the SMARTEN system can be easily changed to other cluster-based techniques. One such variation could be the BERTopic [15] algorithm. Thus, we introduce a comparison with BERTopic [38], and evaluate both algorithms on the searches used by participants in terms of the mean $C_v$ [48] coherence measure for all participant searches. The average runtime of each algorithm across participant searches is also logged, with both models running in a CPU-only manner to ensure comparability. The CPU used in this analysis was an *AMD Ryzen 9 3900X*. We present these predictions in Table 6, indicating that Top2Vec, provides a slight improvement in coherence compared to BERTopic based on the mean value across all particpant searches. Further to this, the average runtime for the Top2Vec approach is considerably less, which presents particular value to the nature of the SMARTEN system, where it is beneficial to provide a minimal wait time for users of the system seeking to perform their analyses.

**Table 6:** Average $C_v$ coherence measures and average runtime in seconds for Top2Vec and BERTopic algorithms across all participant searches

Topic Model	$\boldsymbol{C}_{\boldsymbol{v}}$ Coherence	Runtime (s)
Top2Vec	**0.58**	**5.66**
BERTopic	0.53	20.14

### Qualitative responses from participants

In addition to the quantitative analysis of user perceptions, qualitative feedback regarding the user experiences with the SMARTEN system were also obtained, and presented in Tables 7, 8 and 9 involving *positive aspects*, *aspects that could be improved*, and *general suggestions*, respectively.

**Table 7:** Qualitative responses outlining positive aspects of the SMARTEN system

	Responses to Positive Aspects of SMARTEN
1.	I came across unexpected related words and expanded my knowledge. I could broaden my understanding about related keywords.
2.	I was able to learn more about the strength of relationships between surrounding topics and relationships that are of interest to other researchers.
3.	Knowing the publication year of the paper is useful.
4.	The map is very good.
5.	It was nice that the map was easy to see. English was not a big deal because the keywords were simple.
6.	You can narrow down the field you want to search, using labelling and graphs.
7.	The visualisation is enabled in the system.

**Table 8:** Qualitative responses outlining negative aspects of the SMARTEN system

	Responses to Negative Aspects of SMARTEN
1.	I struggled with English. I would like a Japanese version.
2.	When moving a relationship graph three-dimensionally, it was inconvenient that you could not move it unless you held a circle. I would like to be able to set the centre of the entire graph and move the ball based on that. I want it to be able to rotate no matter where I hold it.
4.	If I type a keyword in Japanese, I want it to be automatically converted to English.
5.	I can’t see the input labels because the input area was too narrow. TOPIC sometimes was busy.
6.	I want several words to be suggested by the system as possible labels and then the user could choose some of them.
7.	Is the labelling process necessary?
8.	It is difficult to input labels.
9.	Labelling is a hassle, and I want about 3 keyword searches.
10.	English was a little bit difficult. I hope at least the contents of the website would be in Japanese.

**Table 9:** Qualitative responses outlining general suggestions for the system improvement

	Responses to General Suggestions after using SMARTEN
1.	Labelling is a hassle.
2.	I would like to see a Japanese version implemented.
3.	I would like to receive a reminder email when a new paper is published in an area that I have searched for.
4.	I think it has great potential as a search tool. I hope it will be updated.

From a *positive perspective* of the SMARTEN system, most participants reported encountering serendipitous related publications while conducting their literature search task, especially one highlighting the system’s efficacy in facilitating the discovery of new knowledge (Table 7, Response 1). Additionally, four participants found the relationship mapping of semantic similarity (Fig. 2) beneficial (Table 7: responses 2, 4, 5 and 6). Furthermore, one participant expressed satisfaction with the publication year graphing feature, which was introduced during the co-design process as described in Co-design of the SMARTEN framework section (Table 7, Response 3). One participant mentioned that the labelling and graphs helped them focus their literature searches(Table 7: responses 6); this response encompasses not only the relationship mappings graph (Fig. 2) but also the topic feature graphs (Fig. 4a). As for response 7, the expert appreciated the various levels of visualisation that are available in the SMARTEN system; it is to be noted that this particular expert did not focus on one specific visualisation, but mentioned it as a generic positive feature.

With regards to *aspects that could be improved* in the SMARTEN system and the *general feedback*, participants identified several areas, mainly concerned with language version, topic labelling, semantic relationship mapping and update notification. Language support functions were proposed by four participants to overcome the language barrier. Regarding topic labelling, five participants stated that they found some difficulties with using the labelling system (responses 5, 6, 8 and 9 in Table 8 and response 1 in Table 9). This was reported as being caused by the graphical interface when labelling publications, particularly due to the input text box (as seen in Fig. 4b) being too small when entering a long list of keywords. Some participants mentioned in their feedback to provide only MeSH category keywords relevant to their search terms to create more space for this input text box. Additionally, one participant suggested that functionality to generate labelling recommendations automatically would be beneficial. One participant stated that they found difficulties with using the relationship mapping functionality (detailed in Semantic similarity of identified topics section, response 2 in Table 8), specifically due to them finding the moving of the graph inconvenient. One participant stated that they would like to have options to increase the number of keyword groups, instead of the current limit of 2 keyword groups during the experiment setting (Table 8, response 9). Moreover, a participant suggested an additional feature of receiving email notifications for newly published papers in their specified area of interest (Table 9, response 3). Lastly, a participant expressed overall satisfaction with the SMARTEN implementation (Table 9, response 4) and expected its updated version.

As shown in Table  10, a thematic analysis of qualitative user feedback was performed to summarise both the strengths of the SMARTEN system and the primary areas for improvement. First, and most significantly, users reported highly positive experiences in (1)Visualisation and Interaction, where users praised the graph’s clarity but requested more intuitive manipulation controls, and (2) Knowledge Discovery, praising the system’s ability to surface unexpected related keywords, clarify conceptual relationships, and provide useful temporal context. Secondly, regarding Language and Localisation, the users expressed a strong demand for Japanese language (their first language) version. This suggests that future work needs to prioritise the development of a multi-language interface. Thirdly, regarding cognitive effort, where the labelling process was identified as a significant friction point, future development should consider providing auto-suggested labels and conducting a comparative evaluation of labelling accuracy between the system with and without the auto-suggested labels. Finally, four feature requests were highlighted for future development. In summary, the qualitative feedback confirms the system’s core value while pinpointing specific enhancements to its usability and accessibility.

**Table 10:** Thematic analysis of user feedback

Theme	Sub-theme	Example comment	Implication
UI	Visualisation & Interaction	T6-4, T6-5, T6-6, T6-7	Consider adding more explicit visual cues for “relationship strength” (e.g., line thickness, statistical measures)
	Language & Localisation	T7-1, T7-10, T8-2	Prioritise development of a multi-language interface.
	Layout of Input component	T7-5	Redesign the input component
UX	Knowledge Discovery & Insight	T6-1, T6-2, T6-3, T6-6	
	Cognitive effort of Labelling Process	T7-7, T7-8, T7-9, T8-1	Consider providing auto-suggested labels or making it optional.
Feature Requests	Alert Systems	T8-3	
	Input Translation	T7-4	
	Auto-suggested labels	T7-6	
	Multiple keyword searches	T7-9	

## Discussion and responses to the research questions

In addressing the first research question regarding the SMARTEN framework, we investigated the application of the Top2Vec [5] algorithm, for the exploratory analysis of medical literature. Compared to conventional probabilistic models such as LDA [24], our framework offers distinct advantages: it enables the automatic detection of the optimal number of topics while facilitating the integration of up-to-date embedding models, which can be easily adapted or updated. This constitutes a practical solution for assisting medical experts, as it lowers the analytical knowledge barrier documented by [9] and streamlines the literature review workflow. Furthermore, the cluster-based topic modeling component in the SMARTEN framework is designed to be extensible. As shown in Table 6, a comparative evaluation with alternative algorithm BERTopic [38] confirms that Top2Vec not only achieves slightly higher topic coherence but also operates with significantly shorter runtime. This combination of effectiveness and speed is critical for minimising wait time in an interactive system, thereby validating our choice of Top2Vec for this study.

While the integration of a clustering-based methodology into topic modeling presents advantages over traditional techniques by facilitating the automatic identification of topics and their comparison via cosine similarity measures, enabling the visualisation of topic relationships such as those illustrated in Fig. 2, it is important to acknowledge that this approach presupposes that documents can be associated with only one topic. In reality, medical research is multifaceted and may encompass multiple themes or topics. Consequently, the topics may not consistently correspond to medical intuition or may seem “superficial” in their thematic representation. For instance, topics may be formed around a specific medication, grouping all literature that refers to that medication together, even though they may discuss entirely disparate themes.

Through the co-design process engaged with domain experts, we have expanded upon our prior research [3, 49] , by creating a comprehensive automatic biomedical literature review framework, with features such as functionality for accounting for aliases and synonyms during literature search, grouping of topics within extracted literature, and presentation of meta-information extracted from publications for identified topics. The resulting SMARTEN system permits analysis of literature based on user-defined queries, which, whilst applied to a given domain, may be extended to others, thus defining an end-to-end solution for literature analysis. The user responses to questions of technology acceptance and general satisfaction after using the SMARTEN system demonstrate that the proposed methodology, through the use of Top2Vec for literature analysis, and subsequent extension through visualisation, has had a positive effect on the discovery of new knowledge for medical experts. Especially, when considering the positive aspects of the system provided by participants, 3 participants highlighted that the visualisation of the relationship map (Fig. 2) was very useful for their study, which is consistent with the result presented in Table 2.

The second research question regarding the perception of users from a medical background when interacting with the SMARTEN framework is answered by the following results: First, the result of the cognitive load analysis shown in Table 1, which suggests that the literature analysis activities during the experiment imposed a moderate cognitive load on users, though with significant variability in their experiences. Second, the result of the technology acceptance analysis, as shown in Table 2, indicates that most participants could easily grasp the operation of the SMARTEN system and acknowledge its usefulness. Finally, the analysis of the general satisfaction, as shown in Table 3, reveals that participants widely agreed that the SMARTEN system effectively supported deeper comprehension of research topics and literature analysis, fostered confidence in understanding new medical subjects, and was worthy of recommendation to colleagues.

Additionally, some participants commented that an interface that presents their first language would be preferred when using SMARTEN. Furthermore, some participants highlighted areas of improvement concerning the interface of the SMARTEN system. Other suggestions included the automatic recommendation of labels, to alleviate the workload of the user during the labelling stage and a notification functionality for finding out when new research is published related to their search.

The third research question, regarding the accuracy of applying Top2Vec as a method for the discovery of literature topics in the SMARTEN framework, is answered through the analysis of labels provided by the users which indicates that an average of $52.3\%$ of suggested publications were relevant to the user’s analysis goal, as shown in Table 4. This varies considerably between users, some users finding $100\%$ of papers suggested to be relevant, compared to as low as $15\%$.

It is worth noting that as a method of identifying relevant papers, an average User-Agreed Relevance of $52.3\%$ indicates that on average, 5 out of 10 suggested papers were deemed as relevant by participants in the topics they chose to analyse. This indicates that the SMARTEN system is beneficial in helping to discern valuable information from the wealth of literature which was identified during the literature searches. When taking into account the lowest labelling accuracy of $15\%$, this still indicates at the lowest as 1 out of the 10 papers as being relevant to the participant’s search. Moreover, as presented in Fig. 5 and Table 5, it is apparent that SMARTEN can enable users to discover literature which would normally be lower-ranked in PubMed, which can assist in the discovery of new knowledge.

In summary, by allowing users to filter topics within the literature based on the topic-words identified by the topic modelling algorithm, and then presenting users with the 10 most relevant publications to that topic, we argue that the SMARTEN framework allows the efficient consolidation and filtering of literature to assist in knowledge discovery from a large volume of publications that could not have been manually analysed. This is also supported by the results of the technology acceptance questionnaire (Table 2), which suggests most participants believed the main system functions were useful for improving their literature analysis and the result of the general satisfaction questionnaire (Table 3) which suggests most participants believed the system enhances the effectiveness of literature analysis and supports learning in medical subjects.

## Study limitations

The interpretation of this study’s findings must take into account its limitations. First, the sample size ($n=18$), while sufficient for an in-depth, qualitative exploration of user perceptions and for identifying strong effect sizes, limits the generalisability (external validity) of the quantitative findings. Recruitment from a single academic center, while ensuring a coherent user group familiar with the domain task, also limits the transferability of results to other institutional contexts. Thus, Subsequent work should involve expanding recruitment to a multi-center, multi-specialty trial to substantiate these preliminary findings within a broader context. Moreover, in future work, we will enhance our system to collect and explore more user behaviour data, such as the time to find a relevant paper, etc.

Secondly, the initial literature retrieval process for SMARTEN is dependent on the PubMed API. Although PubMed is a widely utilised and authoritative database in the realm of biomedical research, it is important to acknowledge the impact of its indexing policies, ranking algorithms, and scope of coverage, which may lead to sampling bias. As a result, the representativeness of the literature retrieved and the subsequent efficacy of the framework are linked to PubMed’s retrieval mechanisms. To address this, future research could expand the framework to incorporate additional sources of medical literature.

Additionally, while we have applied a shallow comparison of Top2Vec with the alternative topic model BERTopic, a deeper analysis of the influence of the ranking through an ablation study, compared with PubMed results, and other established topic modelling techniques could highlight the influence of the proposed framework on knowledge discovery.

## Conclusion and future work

In this paper, we have presented a novel *SMARTEN* framework which is intended to assist medical experts during literature analysis. In particular, we demonstrate the feasibility of the proposed framework in a software implementation, the SMARTEN system. By conducting a study with 18 medical experts about their experience when using the SMARTEN system and examining the feedback and user logs of the participants, we demonstrate that medical professionals generally accepted the framework as a useful tool in their literature analysis, with most participants highlighting that the framework enabled the discovery of new knowledge. Still, areas of improvement remain, with constructive suggestions from participants indicating novel areas of exploration in improving the SMARTEN framework.

The results of our study present several avenues which can be explored to improve the SMARTEN system. Comments in the questionnaire proposed that additional data sources would be beneficial to users, stating that Google Scholar and related repositories should be included. As the general algorithmic approach is robust, it would be possible to apply the SMARTEN framework in a more general setting, and even adjust the framework application, towards allowing analysis of any academic domain, provided that suitable data sources could be configured.

## Supplementary Information

Supplementary Material 1.

## Data Availability

The datasets analysed during the current study are available from the corresponding authors on reasonable request. All data generated during this study are included in this published article and its supplementary information files.

## Abbreviations

SMARTEN: aSsisted MedicAl LiteRaTurE aNalysis
ML: machine learning
AI: artificial intelligence
LDA: Latent Dirichlet Allocation
BERT: Bidirectional Encoder Representations from Transformers
HDBSCAN: Hierarchical Density-Based Spatial Clustering of Applications with Noise
MeSH: Medical Subject Headings
XML: eXtensible Markup Language
UMAP: Uniform Manifold Approximation and Projection
