# Supplementary material for: SMARTEN: a human-AI hybrid framework for assisted medical literature analysis and its evaluation
Source: BMC Med Educ. 2026 Feb 18;26:483. doi: 10.1186/s12909-026-08837-0 (PMC13020293; doi:10.1186/s12909-026-08837-0)
Supplement: Supplementary file 1 — Supplementary Material 1. [file 12909_2026_8837_MOESM1_ESM.docx]

**Appendix**

**Table A1:** Questions Addressing Cognitive Load.

| **Question** | |
| --- | --- |
| 1 | How much effort was required to understand the purpose of using this system? |
| 2 | How much effort was required to label topics, based on the information provided by the system? |
| 3 | When viewing the graphs on the topic details page, how much does the system distract you from finding relevant information? |
| 4 | When viewing the overall topic details page for labelling topics, how discouraged, stressed or irritated did you feel? |

**Table A2:** Questions Addressing Technology Acceptance.

| **Question** | |
| --- | --- |
| 1 | The following feature graphs were easy to use. (This question was followed with Figure 4(a)) |
| 2 | The above feature graphs were useful. |
| 3 | The labelling of the publication list is simple to use. (This question was followed with Figure 4(b)) |
| 4 | The labelling based on publication list was useful. |
| 5 | The following relations map which can display the relevant papers was easy to use. (This question was followed with Figure 2.) |
| 6 | The above relation map which can display the relevant papers was useful. |
